# Digital color-coded molecular barcoding reveals dysregulation of common FUS and FMRP targets in soma and neurites of ALS mutant motoneurons

**DOI:** 10.1038/s41420-023-01340-1

**Published:** 2023-01-26

**Authors:** Maria Giovanna Garone, Debora Salerno, Alessandro Rosa

**Affiliations:** 1 Department of Biology and Biotechnologies “Charles Darwin”, Sapienza University of Rome, Rome, Italy; 2 Center for Life Nano- & Neuro-Science, Fondazione Istituto Italiano di Tecnologia (IIT), Rome, Italy; 3 Laboratory Affiliated to Istituto Pasteur Italia-Fondazione Cenci Bolognetti, Department of Biology and Biotechnologies “Charles Darwin”, Sapienza University of Rome, Rome, Italy; 4 Present Address: Department of Stem Cell Biology, Murdoch Children’s Research Institute, The Royal Children’s Hospital, Parkville, Melbourne, Vic Australia; 5 Present Address: Department of Molecular Medicine, Sapienza University of Rome, Rome, Italy

**Keywords:** Transcriptomics, Amyotrophic lateral sclerosis, Mechanisms of disease

## Abstract

Mutations in RNA binding proteins (RBPs) have been linked to the motor neuron disease amyotrophic lateral sclerosis (ALS). Extensive auto-regulation, cross-regulation, cooperation and competition mechanisms among RBPs are in place to ensure proper expression levels of common targets, often including other RBPs and their own transcripts. Moreover, several RBPs play a crucial role in the nervous system by localizing target RNAs in specific neuronal compartments. These include the RBPs FUS, FMRP, and HuD. ALS mutations in a given RBP are predicted to produce a broad impact on such delicate equilibrium. Here we studied the effects of the severe FUS-P525L mutation on common FUS and FMRP targets. Expression profiling by digital color-coded molecular barcoding in cell bodies and neurites of human iPSC-derived motor neurons revealed altered levels of transcripts involved in the cytoskeleton, neural projection and synapses. One of the common targets is HuD, which is upregulated because of the loss of FMRP binding to its 3′UTR due to mutant FUS competition. Notably, many genes are commonly altered upon FUS mutation or HuD overexpression, suggesting that a substantial part of the effects of mutant FUS on the motor neuron transcriptome could be due to HuD gain-of-function. Among altered transcripts, we also identified other common FUS and FMRP targets, namely *MAP1B*, *PTEN*, and *AP2B1*, that are upregulated upon loss of FMRP binding on their 3’UTR in FUS-P525L motor neurons. This work demonstrates that the impairment of FMRP function by mutant FUS might alter the expression of several genes, including new possible biomarkers and therapeutic targets for ALS.

## Introduction

RNA-binding proteins (RBPs) control RNA metabolism and proteome in neurons. These functions are crucial for proper axon and dendrite development, guidance, targeting, and synapse formation. Several RBPs, including FUS, are mutated or aggregated in amyotrophic lateral sclerosis (ALS), a neurodegenerative disease primarily caused by the death of motor neurons (MNs) [1, 2]. Most ALS-associated FUS mutations are missense mutations in the nuclear localization signal, resulting in protein mislocalization to the cytoplasm [3–5]. In neuronal cells, FUS localizes in specific compartments such as the neuromuscular junction [6], presynaptic terminals [7, 8], and post-synaptic dendrites [9–12], where it acts as local translation regulator [12, 13] and is responsible for the synaptic structure and function [11, 14].

We previously showed that genes whose transcripts are bound in the 3′UTR by mutant FUS show altered protein levels and are involved in cytoskeleton and neuron projection [15, 16]. This suggests that changed targeting by cytoplasmic mutant FUS might promote axonopathy. Indeed, axonal dysfunction has been reported in early symptomatic ALS patients and it occurs before the motor phenotype in animal models [17, 18], indicating that ALS can be classified as a distal axonopathy due to alteration in the neuronal cytoskeleton, RNA transport, axonal energy supply, clearance of junk proteins, and aberrant axonal branching [19, 20]. To this regard, we recently reported increased axon branching and arborization, as well as faster outgrowth after injury in FUS-ALS MNs, due to increased activity of the neuronal RBP HuD (ELAVL4) [21].

HuD is involved in neuron development, synaptic plasticity, and response of peripheral neurons to damage [22–24]. We have shown competition between ALS mutant FUS and the translational repressor FMRP for HuD 3′UTR binding, resulting in increased HuD levels, with consequences on HuD targets, NRN1 and GAP43, in turn involved in abnormal axonal morphology and recovery upon injury [15, 21, 25]. Notably, similar axonal phenotypes have been reported also in non-FUS ALS models [26, 27], and upregulated HuD levels were observed in sporadic ALS patients [28]. These evidences suggest a possible transversal role of HuD in ALS.

Here, we took advantage of iPSC-derived MNs and RNA profiling by digital color-coded molecular barcoding in soma and neurites to gain insight into the molecular mechanisms underlying axonal dysfunctions in ALS, with a specific focus on the interplay between three RBPs: ALS mutant FUS, FMRP and HuD. We found altered expression of neurodevelopment, cytoskeleton and synapse genes upon FUS mutation, FMRP loss or HuD overexpression. Among them, we identified genes previously involved in nerve growth, arborization and regeneration, in physiological and pathological conditions, such as *MAP1B*, *AP2B1*, and *PTEN*, which represent new common FMRP and FUS^P525L^ targets [29–31]. Translation of *MAP1B*, *AP2B1*, and *PTEN* is negatively controlled by FMRP in FUS wild-type MNs and increased in FUS mutant MNs. This work suggests that the intrusion of mutant FUS into FMRP functions might exert a broad impact on the transcriptome of both neurites and soma in ALS MNs.

## Results

### Identification of common FMRP and FUS targets

We have previously identified the transcripts bound by FUS in iPSC-derived human MNs, showing that while FUS^WT^ preferentially binds introns, the ALS mutant FUS^P525L^ interacts with 3′UTRs [15]. Thus, the consequences of FUS pathological mutations might arise by both loss-of-function (loss of intron binding due to reduced nuclear FUS levels) and gain-of-function (gain of 3′UTR binding due to increased accumulation of FUS in the cytoplasm) mechanisms. Changes in FUS mRNA targets correlated with changes in the proteome [16]. Moreover, we discovered competition between mutant FUS and FMRP for the binding to HuD 3′UTR, resulting in the loss of negative regulation by FMRP on HuD translation [21].

**Fig. 1:**
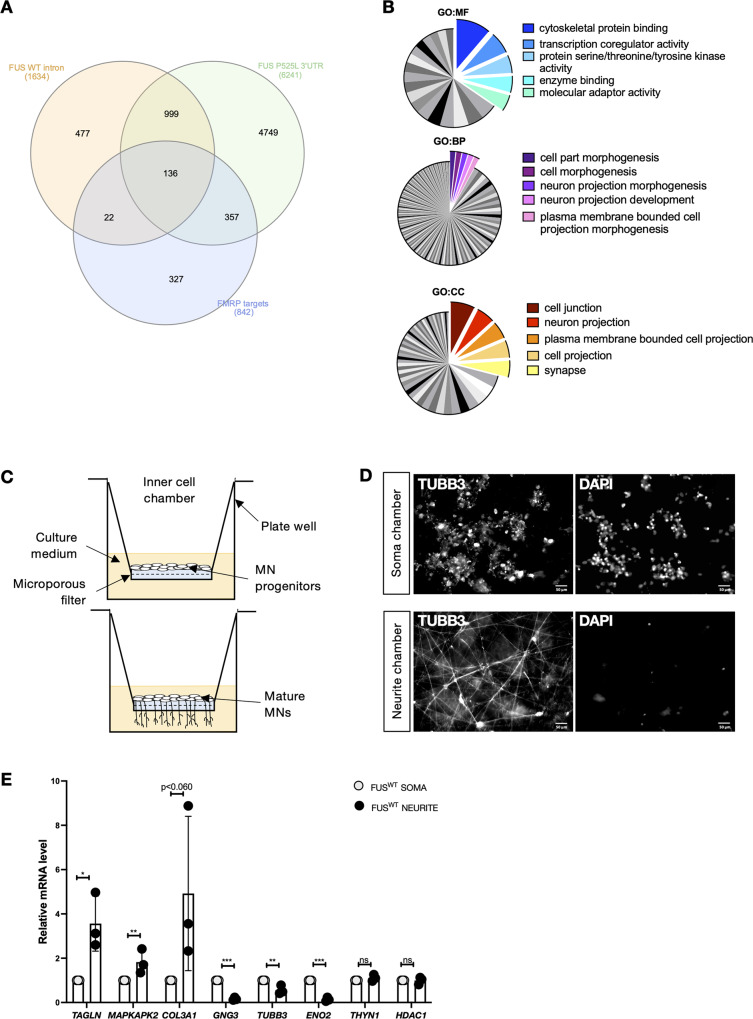
FMRP and FUS interactomics analyses and neuron cell culture system. **A** Venn diagram showing the overlap among the transcripts bound in intronic regions by FUS^WT^ (*p*-value < 0.05) and in the 3′UTR by endogenous FUS^P525L^ (*p*-value < 0.05) and FMRP targets (*p*-value < 0.05). **B** GO term enrichment analysis of the 136 overlapped genes. The −log10 (adjusted *p*-value) associated with each category is represented by the size of the pie slice. GO:MF (molecular function); GO:BP (biological process); GO:CC (cellular component). **C** Schematic representation of dissociated and re-plated iPSC-derived spinal MN progenitor cells at day 5 of differentiation (top) and mature MNs (bottom) into cell culture insert. **D** Immunostaining of TUBB3 and staining with DAPI in MNs cultured in cell culture insert. Scale bar: 50 µm. **E** Analysis of the mRNA levels of the indicated genes by real-time qRT-PCR in iPSC-derived spinal MNs. *TAGLN*, *MAPKAPK2*, *COL3A1* are neurite markers; *GNG3*, *ENO2* are neuronal cell body enriched markers; *THYN1*, *HDAC1* are housekeeping genes, included in this analysis as uniformly distributed transcripts. The graph shows the average from three independent differentiation experiments, error bars indicate the standard deviation (Student’s *t*-test; paired; two tails; *p < 0.05; **p < 0.01; ***p < 0.001; n.s. non significant).

These observations prompted us to assess if ALS FUS mutation might interfere with FMRP activities on other relevant genes, including genes that might be affected at the level of alternative splicing by loss of FUS^WT^ and genes targeted in their 3′UTR by FUS^P525L^. The possible common targets of FMRP and FUS were identified from previously published FUS PAR-CLIP [15] and FMRP HITS-CLIP [32] data. We found a common set of 1135 targets bound both by FUS^WT^ in intronic regions and by FUS^P525L^ in the 3′UTR (Fig. 1A). Cross-reference with FMRP HITS-CLIP data, comprising FMRP interacting mRNAs whose translation is inhibited by delaying ribosomal translocation at a *p*-value < 0.05, identified 136 common targets (Fig. 1A) (Supplementary Table S1). Gene ontology (GO) term enrichment analysis of this subset revealed categories mainly related to cytoskeletal protein binding (molecular function, GO: MF), neuron projection development and morphogenesis (biological process, GO: BP), neuron projection, plasma membrane bounded cell projection, synapse (cellular component, GO: CC) among the top five terms (Fig. 1B; Supplementary Table S2), suggesting that common FMRP and FUS targets include genes that play crucial roles in subcellular neuronal compartments.

Taken together these data suggest that the FUS mutation might impinge the expression levels, splicing and/or subcellular localization of several FMRP mRNA targets. This evidence prompted us to analyze the levels of common FMRP and FUS mRNA interactors in soma and neurite compartments.

### Inner chamber iPSC-derived MN cultures to analyze gene expression in soma and neurites

In order to isolate the RNA from different cellular compartments without cross-contamination, we took advantage of modified Boyden chambers, which have been previously used to study the axonal transcriptome of mouse embryo dorsal root ganglia [33]. Human iPSCs were converted into spinal MN progenitors (day 5) with a protocol that allows the production of nearly pure motoneuronal population without the need of cell sorting [34, 35] and re-plated inside the inner chamber onto a porous membrane that enabled axons to grow across the filter, restricting the cell bodies to the top membrane surface (Fig. 1C). Correct compartmentalization of soma and neurites was assessed by neuronal tubulin immunostaining and nuclei labeling at day 12 of differentiation (Fig. 1D). RNA samples collected from soma and neurites were then analyzed by quantitative RT-PCR. We observed enrichment of neuronal projections markers *TAGLN*, *COL3A1*, and *MAPKAK2* in the neurite compartment, and enrichment of *GNG3* and *ENO2* in the soma (Fig. 1E), consistent with the known localization of these transcripts in neurons [36].

### Single-mRNA molecules detection of FMRP and FUS targets in soma and neurites

The FUS and FMRP common targets were examined based on their known role in neurodevelopmental and/or neurodegenerative disorders by taking advantage of the DISEASES database [37]. Among the 136 FMRP targets bound both by FUS^WT^ in intronic regions and by FUS^P525L^ in the 3′UTR, 70 candidates with the highest z-score (Supplementary Table S3) were selected for gene expression analysis in the soma and axonal compartments. In consideration of previous findings [21], we added to this list HuD (*ELAVL4*) and its target genes, *NRN1* and *GAP43*. Transcripts quantification was performed by NanoString digital color-coded molecular barcoding, which employs fluorescent barcodes for the direct detection of distinct mRNA targets in a single run without amplification, with a sensitivity of 1 copy per cell and requiring nanoscale amounts of RNA [38]. A code set specific to a 100-base region of the target mRNA was designed using a 3′ biotinylated capture probe and a 5′ reporter probe tagged with a specific fluorescent barcode, creating two sequence-specific probes for each target transcript (Supplementary Table S4).

**Fig. 2:**
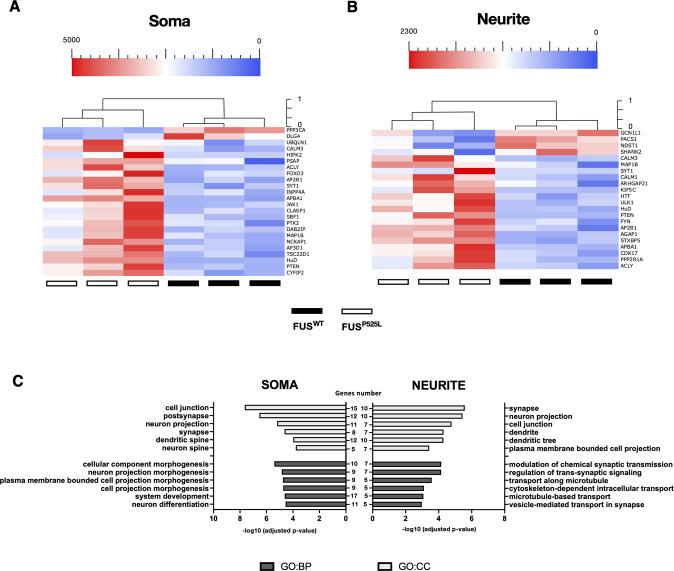
Differentially expressed genes in soma and neurite of FUS^WT^ and FUS^P525L^ MNs. **A**, **B** Unsupervised cluster representing the RNA molecules count of differentially expressed genes in the cell body (**A**) or neurite (**B**) compartment of iPSC-derived FUS^WT^ and FUS^P525L^ MNs. Plotted values correspond to normalized RNA molecules as described in the Methods section. Red, higher abundance; blue, lower abundance. **C** Bar graph representation of the top 6 GO terms enriched in differentially expressed genes as in (**A**, **B**). The −log10(adjusted *p*-value) and number of genes are shown.

**Fig. 3:**
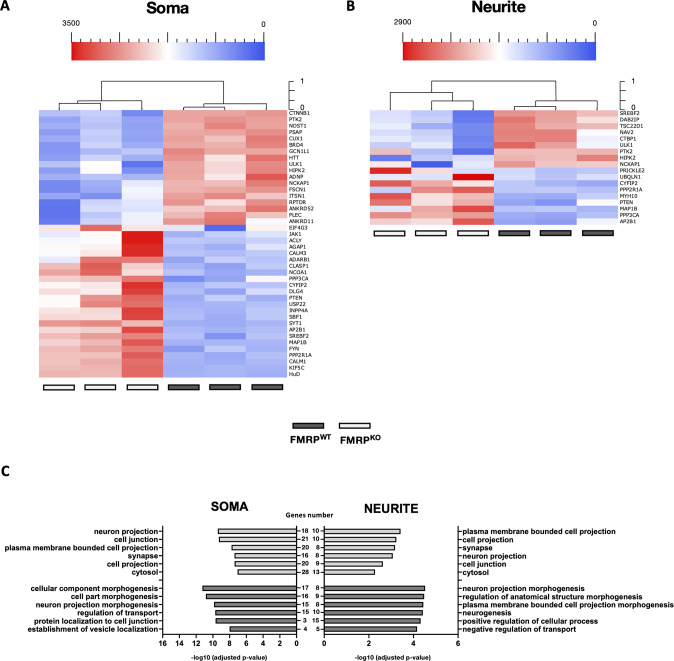
Differentially expressed genes in soma and neurite of FMRP^WT^ and FMRP^KO^ MNs. **A**, **B** Unsupervised cluster representing the RNA molecules count of differentially expressed genes in the cell body (**A**) or neurite (**B**) compartment of iPSC-derived FMRP^WT^ and FMRP^KO^ MNs. Plotted values correspond to normalized RNA molecules as described in the Methods section. Red, higher abundance; blue, lower abundance. **C** Bar graph representation of the top 6 GO terms enriched in differentially expressed genes as in (**A**, **B**). The −log10(adjusted *p*-value) and number of genes are shown.

MNs differentiated from an isogenic pair of FUS^WT^ and FUS^P525L^ iPSCs [39] were cultured in modified Boyden chambers to allow RNA isolation from soma and neurites. We identified 24 (soma) and 22 (neurites) targets that were differentially expressed in FUS^P525L^ MNs (Fig. 2A, B). Interestingly, over 80% of gene expression changes consisted of overexpression. We performed GO enrichment analysis to characterize the biological process and cellular component of these genes (Fig. 2C, Supplementary Table S5). We found that targets altered in the neurite subcellular compartment showed enrichment in biological processes involving “regulation of trans-synaptic signaling”, “transport along microtubule”, “cytoskeleton-dependent intracellular transport”, and cellular components related to “synapse”, “neuron projection”, “cell junction”, and “dendrite” (Fig. 2C). On the other hand, altered targets in the cell body fraction are involved in nervous “system development”, “neuron projection morphogenesis”, “neuron differentiation”, and enriched for “cell junction”, “postsynapse” and “neuron projection” compartment (Fig. 2C). Together, these data suggest that the FUS mutation might impact the synaptic component in axons and affect the function of genes in the soma involved in neuron development and morphogenesis. The same analysis in MNs derived from isogenic FMRP^WT^ and FMRP^KO^ human iPSCs [40] resulted in 42 differentially expressed targets in the soma and 18 in neurites (Fig. 3A, B). For these genes, GO enrichment analysis revealed categories related to the regulation of transport and localization, and regulation of cellular processes and development in both compartments (Fig. 3C, Supplementary Table S5). These data support the role of FMRP as an essential RBP for transporting mRNAs to subcellular neuron sites with implications in neurodevelopmental diseases.

### Increased HuD levels in MNs mimics the effects of ALS mutant FUS

**Fig. 4:**
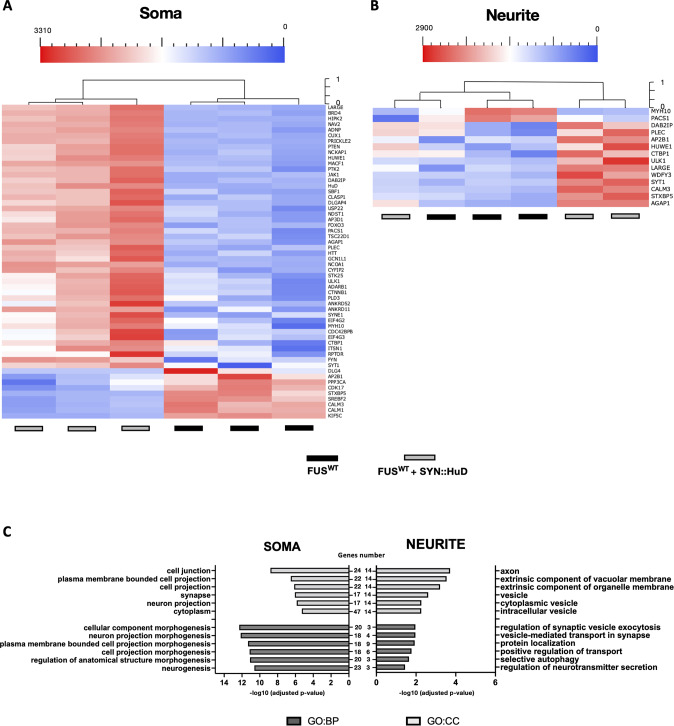
Differentially expressed genes in soma and neurite of FUS^WT^ and FUS^WT^ SYN::HuD MNs. **A**, **B** Unsupervised cluster representing the RNA molecules count of differentially expressed genes in the cell body (**A**) or neurite (**B**) compartment of iPSC-derived FUS^WT^ MNs and MNs derived from a stably transfected FUS^WT^ iPSC line expressing a HuD transgene under the control of the neuronal-specific human synapsin 1 promoter (SYN1::HuD). Plotted values correspond to normalized RNA molecules as described in the Methods section. Red, higher abundance; blue, lower abundance. **C** Bar graph representation of the top 6 GO terms enriched in differentially expressed genes as in (**A**, **B**). The −log10(adjusted *p*-value) and number of genes are shown.

**Fig. 5:**
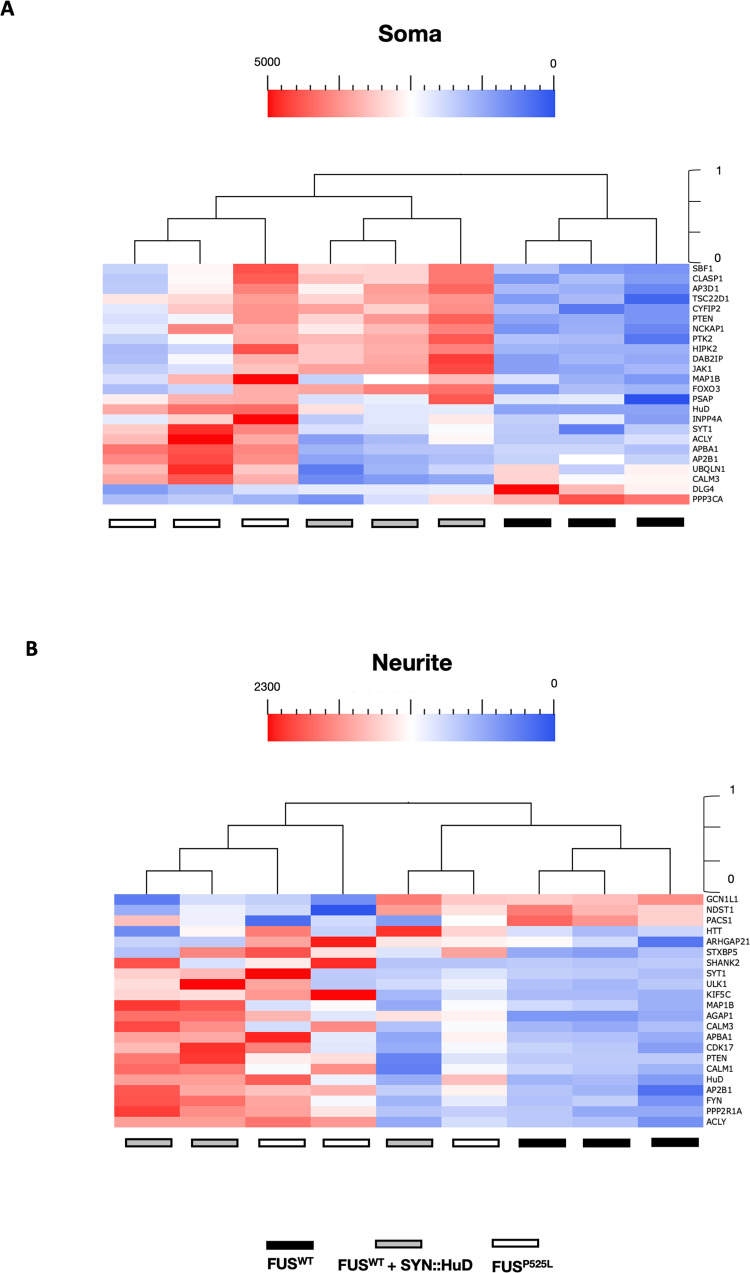
Differentially expressed genes in soma and neurite of FUS^WT^, FUS^P525L^, and FUS^WT^ SYN::HuD MNs. **A**, **B** Unsupervised cluster representing the RNA molecules count of differentially expressed genes in the cell body (**A**) or neurite (**B**) compartment of iPSC-derived FUS^WT^, FUS^P525L^, and FUS^WT^ SYN::HuD MNs. Plotted values correspond to normalized RNA molecules as described in the Methods section. Red, higher abundance; blue, lower abundance.

We found increased HuD levels in neurites and soma of FUS^P525L^ and in the soma of FMRP^KO^ MNs (Figs. 2 and 3), confirming our previous observation [21]. Thus, changes in the transcriptome in both these genetic backgrounds might be due, to some extent, to HuD increased levels. To experimentally address this possibility, we performed single-mRNA molecules detection in HuD overexpressing MNs, with an otherwise FUS^WT^ and FMRP^WT^ background. To this aim, we took advantage of a stably transfected iPSC line expressing a HuD transgene under the control of a neuronal-specific promoter (SYN1::HuD), in which HuD levels were increased in a range similar to that observed in FUS^P525L^ or FMRP^KO^ cells [21]. Fifty-six (soma) and 14 (neurites) genes were differentially expressed upon HuD overexpression (Fig. 4A, B). Also in this case, most of the change was represented by upregulation. GO enrichment analysis showed an interesting overlap with categories resulting from the analysis of FUS^P525L^ MNs. Indeed, altered neurite transcripts were involved in biological processes related to synaptic transmission. On the other hand, neuronal morphogenesis and differentiation terms were enriched in the soma (Fig. 4C, Supplementary Table S5). In agreement with this observation, analysis of differentially expressed transcripts showed that SYN1::HuD biological replicates tend to cluster with FUS^P525L^ samples, rather than with their common parental line (FUS^WT^), both in the soma and neurite compartments (Fig. 5). We also noticed that the levels of several transcripts were altered in both soma and neurites (Fig. S1). In all conditions, most of them were upregulated in both compartments. This is particularly evident for FUS^P525L^, in which all genes analyzed were commonly upregulated in both soma and neurites, while in the case of FMRP^KO^ their levels were commonly increased or decreased. In the case of Syn::HuD, however, we noticed that while the majority of the transcripts were upregulated in both compartments, levels of five transcripts changed in opposite directions: two were increased in the soma and decreased in neurites (suggesting partial impairment in their neuritic localization) and three were increased in neurites and decreased in the soma (suggesting enhancement of their neuritic localization).

Collectively, these results suggest that increased HuD levels might substantially contribute to altered gene expression observed in FUS mutant MNs, in particular for disease-relevant transcripts possibly involved in axopathogenesis. Moreover, HuD upregulation might directly or indirectly alter the subcellular localization of some transcripts.

### ALS mutant FUS competes with FMRP for 3′UTR binding of disease relevant genes

We next focused on common FUS and FMRP targets that, similarly to HuD, were increased in both FUS^P525L^ and FMRP^KO^ MNs and identified three interesting candidates. *AP2B1* (adapter related protein complex 2 subunit beta 1) encodes for a subunit of the adapter protein complex 2 (AP2), involved in cargo selection, vesicle assembly, and recycle of synaptic vesicle membranes from the presynaptic surface [41, 42]. *MAP1B*, a well-known FMRP target, plays an important role in the tyrosination of alpha-tubulin in neuronal microtubules and is involved in the cytoskeletal modifications associated with neurite outgrowth [43]. *PTEN* (phosphatase and tensin homolog) encodes for a protein phosphatase that has been recently implicated in neurodevelopmental and neurodegenerative diseases [44–48]. Increased mRNA levels for these genes in FUS^P525L^ and FMRP^KO^ MNs were validated by qRT-PCR (Fig. S2A and S2B).

**Fig. 6:**
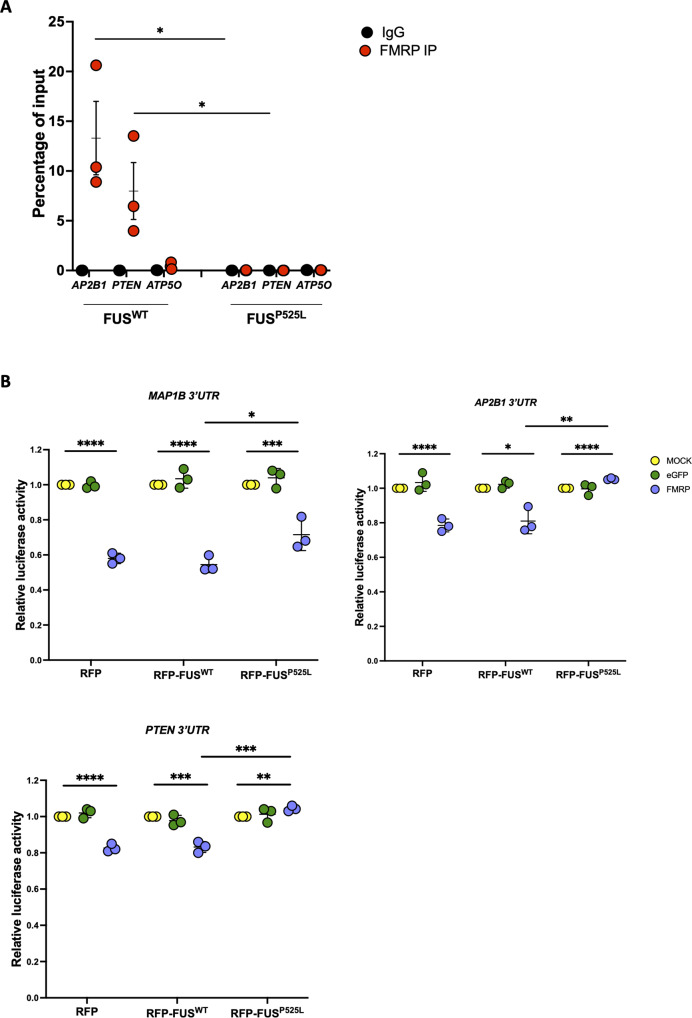
FMRP and FUS^P525L^ compete for *AP2B1*, *PTEN*, and *MAP1B* 3’UTR binding. **A** Analysis of *AP2B1* and *PTEN* mRNA levels by real-time qRT-PCR in FMRP RIP samples from FUS^WT^ or FUS^P525L^ iPSC-derived spinal MNs. The housekeeping gene *ATP5O* is used as negative control. The graph shows the relative enrichment of the mRNAs pulled down by FMRP immunoprecipitation (IP), calculated as the percentage of input, in IP or control IgG samples, after normalization with an artificial spike RNA. The average from three independent differentiation experiments is showed and error bars indicate the standard deviation (Student’s *t*-test; unpaired; two tails; *p < 0.05). **B** Luciferase assay in HeLa cells expressing RFP, RFP-FUS^WT^, or RFP-FUS^P525L^ and transfected with Renilla luciferase reporter constructs containing the 3′UTR of *AP2B1* (RLuc-*AP2B1*-3′UTR), *MAP1B* (RLuc-*MAP1B*-3′UTR) or *PTEN* (RLuc-*PTEN*-3′UTR), in combination with plasmids overexpressing FMRP, eGFP or alone (MOCK) (Student’s *t*-test; paired; two tails; *p < 0.05; **p < 0.01; ***p < 0.001; ****p < 0.0001).

**Fig. 7:**
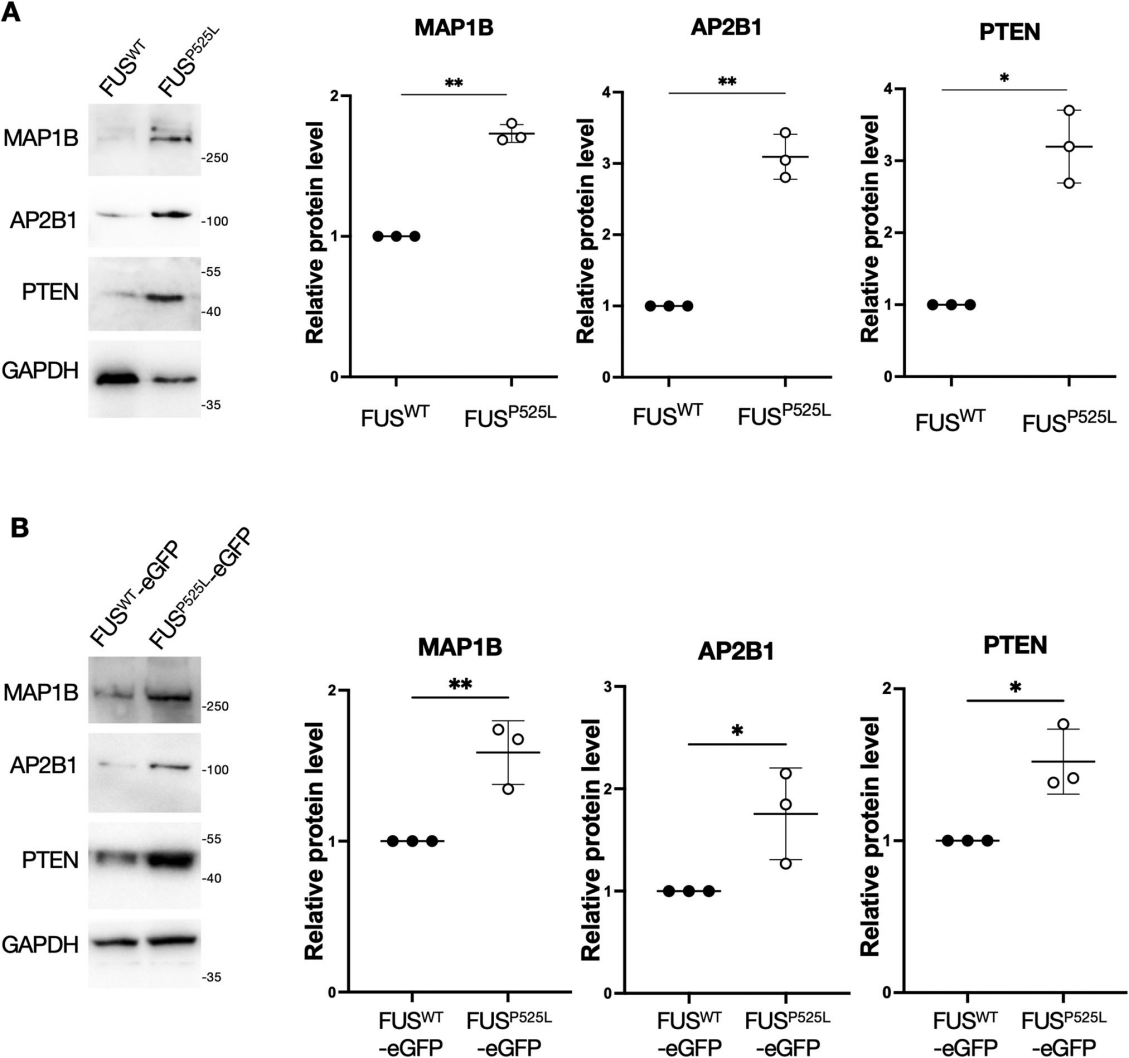
AP2B1, PTEN, and MAP1B protein levels are increased in FUS mutant MNs. **A** Western blot analysis of the indicated genes protein levels in FUS^WT^ and FUS^P525L^ iPSC-derived spinal MNs. The molecular weight (kDa) is indicated on the right. The graphs show the average from three independent differentiation experiments, error bars indicate the standard deviation (Student’s *t*-test; unpaired; two tails; *p < 0.05; **p < 0.01). GAPDH signal was used for normalization. Protein levels are relative to the FUS^WT^ sample for each experiment. **B** Same analysis as in (**A**), performed in MNs from an independent isogenic pair of wild-type and P525L iPSC lines, in which endogenous FUS is fused at the C-terminal to the enhanced green fluorescent protein (eGFP) [49].

FMRP binding to these transcripts in the presence or absence of mutant FUS was evaluated by native RNA immunoprecipitation (RIP). FMRP was immunoprecipitated from human iPSC-derived MN extracts, and the associated mRNAs were analyzed by quantitative RT-PCR. As previously shown for *HuD* and *MAP1B* [21], we observed a significant enrichment of *AP2B1* and *PTEN* in FMRP RIP from FUS^WT^ MNs, while the housekeeping gene ATP5O served as a negative control. Interestingly, FMRP binding to both targets was completely abolished in FUS^P525L^ MNs (Fig. 6A). These data suggest that impairment of FMRP binding to target RNAs in mutant FUS MNs, possibly due to competition for 3′UTR binding, might occur for several common FUS and FMRP targets. To assess the functional consequences of the loss of FMRP interaction in the presence of FUS^P525L^, we took advantage of a reporter assay based on a luciferase gene fused to the 3′UTR of the target of interest. *AP2B1*, *MAP1B*, and *PTEN* 3′UTR reporters were transfected in HeLa cells ectopically expressing a wild-type or P525L mutant FUS transgene (both fused to RFP, or RFP alone as control) in combination with an FMRP transgene or eGFP as control. As shown in Fig. 6B, when FMRP was overexpressed in the presence of RFP alone, the 3′UTR reporter activity was significantly decreased for all the candidates. Notably, co-expression of RFP-FUS^P525L^, but not RFP-FUS^WT^, partially reversed (for *MAP1B*) or completely abolished (for *AP2B1* and *PTEN*) such negative regulation by FMRP. These results suggest that FMRP could act as a negative regulator of *AP2B1*, *MAP1B* and *PTEN* translation by direct 3′UTR binding, while mutant FUS competition would impair such function. We next analyzed protein levels of all three candidates in iPSC-derived MNs, observing significant upregulated levels in mutant FUS as well as FMRP KO cells (Fig. 7A, Fig. S2C). Importantly, AP2B1, MAP1B, and PTEN protein levels were analyzed in MNs derived from an independent isogenic pair of wild-type and P525L iPSC lines [49], confirming this result (Fig. 7B).

Collectively, these results show that *AP2B1*, *MAP1B*, and *PTEN* represent novel common FMRP and FUS targets whose levels are kept low by FMRP in normal conditions, and aberrantly increased in FUS mutant MNs.

## Discussion

FMRP and FUS, genetically linked to Fragile-X syndrome and ALS respectively, are two multifunctional RBPs involved in post-transcriptional gene expression. Notably, changes in the activity of FMRP in FUS-ALS models have been recently reported [21, 50, 51], suggesting compromised FMRP functions with profound effects on the RNA metabolism in MNs. We previously demonstrated that one of the consequences of mutant FUS interference in FMRP functions is HuD upregulation [21]. Here, we show that HuD overexpression, in otherwise normal MNs, produces changes strikingly similar to those observed in mutant FUS MNs on the expression of a set of genes that are: (a) involved in nervous system diseases; (b) targets of wild-type FUS in intronic regions; (c) targets of mutant FUS in the 3′UTR; (d) targets of FMRP; (e) involved in synaptic transmission and neuron development. Moreover, some of these transcripts showed altered subcellular localization upon HuD overexpression. These findings, together with previous evidence of HuD upregulation and localization in pathological cytoplasmic inclusions in familial and sporadic ALS patients [15, 28, 50], support the importance of this factor in the context of a complex regulatory RBP network, which is in place in normal MNs and disrupted in ALS.

We describe here additional common FUS and FMRP targets. Like HuD, regulation of *MAP1B*, *AP2B1* and *PTEN* translation by the two RBPs depends on their 3′UTR. FUS-dependent upregulation of *MAP1B* was previously reported in FUS and Ubiquilin-2 models [50, 52]. Competition between FUS and FMRP for *MAP1B* mRNA binding at a G quadruplex structure in the 5′UTR has been previously proposed [53]. Our new findings suggest that such competition might extend to the 3′-UTR as well.

AP2B1 is involved in clathrin-mediated endocytosis and its knockdown reduces the number of dendrites in developing neurons [30]. Increased levels of AP2B1 have been recently observed in the cerebrospinal fluid of Alzheimer’s disease patients [54]. Our work suggests that this protein might be explored as a possible biomarker in ALS as well.

Increasing evidence links PTEN to neurodevelopmental and neurodegenerative diseases [44, 45]. PTEN translation is negatively regulated by FMRP and heterozygous loss of *Pten* rescued neuronal phenotypes in an *Fmr1* knockout mouse [55]. Moreover, it has been shown that PTEN knockdown or pharmacological inhibition is beneficial for MN survival and neuromuscular innervation in non-FUS ALS and spinal muscular atrophy models [46–48, 56]. Our data suggest that PTEN levels could be increased due to a strong impairment of FMRP binding and activity by mutant FUS, possibly extending the applicability of this therapeutic approach to FUS-ALS. We further noticed that *PTEN* transcript levels were increased, in the absence of FUS or FMRP mutations, in the soma of HuD overexpressing MNs (Fig. 4A). Moreover, in the same cells *AP2B1* localization in neurites was increased (Fig. S1). While we did not find any significant change in the levels of *MAP1B* transcript, HuD had been previously reported to interact at the protein-protein level with the light chain of MAP1B protein [57]. Interestingly, *AP2B1*, *MAP1B*, and *PTEN* transcripts have been previously found among the interactors of neuronal ELAVL proteins [58, 59]. These findings suggest that the expression, localization and/or activity of these targets might be also under the control of HuD.

In conclusion, we propose that the analysis of common FMRP and FUS targets, which are dysregulated in soma and neurites of human MNs, could identify relevant biomarkers and therapeutic targets for ALS.

## Materials and methods

### Cell culture and differentiation

Human iPSC lines used in this study are: FUS^WT^ and FUS^P525L^ (ref. [39]); FMR1 KO [40]; SYN1::HuD [21]; KOLF WT 2 and P525L16 (LL FUS-eGFP) [49]. As indicated in the original studies, informed consent had been obtained from all patients involved prior to cell donation. Cells were regularly tested for mycoplasma contamination. Cells were maintained and induced to differentiate into MNs as described [21]. At day 5, MN progenitors were dissociated with Accutase (Thermo Fisher Scientific, Waltham, MA, USA) and plated on Matrigel (BD Biosciences Franklin Lakes, NJ, USA)-coated dishes or in modified Boyden chambers (Merck, Darmstadt, Germany) 6-well hanging inserts 1.0 µm PET. On day 12, each inner cell culture was washed with PBS w/o Ca^2+^/Mg^2+^ and scraped using a cell lifter to collect the soma compartment. For neurites isolation, the membrane was peeled off with a cutter, pulled into a 2.0 ml tube, lysed with TRK lysis buffer of the Micro Elute Total RNA Kit (VWR International PBI, Milan, Italy) and left rotating on a wheel for 20 min at room temperature before RNA extraction.

### Immunofluorescence

Immunofluorescence was performed with anti-TUJ1 primary antibody (1:1000; T2200; Sigma-Aldrich; RRID:AB_262133) and donkey anti-rabbit Alexa Fluor 488 secondary antibody (1:200; IS20015-1; Immunological Sciences, Rome, Italy) as described [21]. DAPI (1:2000; Merck) was used to stain nuclei. Cells were imaged using an inverted Zeiss LSM 780 microscope.

### Soma and axon compartments RNA profiling

The nCounter custom code set (NanoString Technologies, Seattle, WA, USA) is indicated in Supplementary Table S4. Briefly, 50 ng of RNA was hybridized to the capture and reporter probe sets at 65 °C for 20 h and applied to the nCounter preparation station. Data were collected using the nCounter Digital Analyzer (NanoString Technologies) via counting individual fluorescent barcodes and quantifying target mRNA molecules in each sample performing a high-density scan (555 fields of view-FOV).

### Bioinformatics analysis

The NanoString nSolver 4.0 software (https://www.nanostring.com/products/analysis-software/nsolver) was used to subtract background and normalize the RNA count data using the geometric means of the positive controls and the housekeeping genes *ATP5O* and *THYN*. Background subtraction and data normalization were performed for each independent class comparison. Gene expression was averaged across the three independent replicate samples. GraphPad Prism 6.0 (GraphPad Software, San Diego, CA, USA) was used to determine differentially expressed genes; a Wilcoxon signed-rank test (for two conditions) or one-way ANOVA (for three conditions) was performed on the sample normalized data to determine statistical significance. Unsupervised hierarchical clustering and Gene ontology analysis of differentially expressed genes was performed using Qlucore Omics Explorer software and GO profiler tool, respectively.

PAR-CLIP reads and transitions were derived from published data [15]; only ratio of T to C transitions > 1 (comparing FUS^P525L^ vs. FUS^WT^) were considered for the comparison with the FMRP HITS-CLIP data [32]. Paired Student’s *t*-test was used to determine the alternative and exclusive binding of FUS-WT and FUS^P525L^ on intron and coding sequences, respectively. Intersection between FUS PAR-CLIP and FMRP HITS-CLIP datasets was performed considering only genes detected in both experiments.

### RNA immunoprecipitation

RNA immunoprecipitation was performed on iPSC-derived MNs with 10 µg of anti-FMRP (ab17722; Abcam, Cambridge, UK; RRID:AB_2278530) or rabbit monoclonal anti-human IgG antibody (ab109489; Abcam; RRID:AB_10863040) as described [21]. An artificial spike RNA in vitro transcribed from the pcDNA3.1 plasmid was added to the samples before extraction.

### Real-time qRT-PCR

Total RNA was retrotranscribed with iScript Supermix (Bio-Rad Laboratories, Hercules, CA, USA) and analyzed by real-time qRT-PCR with iTaq Universal SYBR Green Supermix (Bio-Rad Laboratories). *ATP5O* was used as the internal calibrator. Primers sequences are listed in Supplementary Table S6.

### Luciferase assay

3′UTR sequences were cloned in the pSI-Check2 vector (Promega, Fitchburg, WI, USA) using the primers listed in Supplementary Table S6, downstream the hRluc coding sequence. The resulting pSI-Check2-3’UTR constructs were transfected alone or in combination with epB-Bsd-TT-FMR1 or epB-Bsd-TT-eGFP in 5 × 10^4^ pre-seeded HeLa cells expressing RFP, RFP-FUS^WT^ or RFP-FUS^P525L^ as described [21]. Twenty-four hour of post-transfection luciferases activity was measured by Dual Glo luciferase assay (Promega).

### Western blot

Western blot analysis was carried out as described [21] using anti-AP2B1 (1:2000; 15690-1-AP; Proteintech, Rosemont, IL, USA; RRID:AB_2056351), anti-MAP1B (1:1000; 21633-1-AP; Proteintech; RRID:AB_10793666), anti-PTEN (1:2000; 22034-1-AP; Proteintech; RRID:AB_2878977), anti-GAPDH (1:2000; MAB-10578; Immunological Sciences) primary antibodies and HRP Donkey Anti-Mouse IgG (H + L) (IS20404; Immunological Sciences) and HRP Donkey Anti-Rabbit IgG (H + L) (IS20405; Immunological Sciences) secondary antibodies. Uncropped blots are shown in Supplementary Figure S3.

### Statistical analysis

Statistical analysis, graphs, and plots were generated using GraphPad Prism 6 (GraphPad Software). As indicated in each figure legend, Student’s *t*-test or ordinary one-way ANOVA was performed, and data set are shown in dot plots indicating mean ± standard deviation (st.dev.). Sample size and the definition of replicates for each experiment is also indicated in the figure legends. RNA or protein expression was averaged across the three independent replicate samples.

## Supplementary information

Supplementary figures and tables legends

Full lenght uncropped western blots

Supplementary Table S1

Supplementary Table S2

Supplementary Table S3

Supplementary Table S4

Supplementary Table S5

Supplementary Table S6

## Data Availability

All data generated or analyzed during this study are included in the manuscript and supporting files; Source Data files have been provided for all figures in the manuscript. Materials are available upon request.
