# Supplementary material for: Digital color-coded molecular barcoding reveals dysregulation of common FUS and FMRP targets in soma and neurites of ALS mutant motoneurons
Source: Cell Death Discov. 2023 Jan 26;9:33. doi: 10.1038/s41420-023-01340-1 (PMC9879958; doi:10.1038/s41420-023-01340-1)
Supplement: Supplementary file 1 — Supplementary figures and tables legends [file 41420_2023_1340_MOESM1_ESM.pdf]

SUPPLEMENTARY INFORMATION

Digital color-coded molecular barcoding reveals dysregulation of common FUS and FMRP targets in soma and neurites of ALS mutant motoneurons

Maria Giovanna Garone[1,2,~], Debora Salerno[2,#], Alessandro Rosa[1,2,3,*]

[1] Department of Biology and Biotechnologies "Charles Darwin", Sapienza University of Rome, Rome, Italy.

[2] Center for Life Nano- & Neuro-Science, Fondazione Istituto Italiano di Tecnologia (IIT), Rome, Italy.

[3] Laboratory Affiliated to Istituto Pasteur Italia-Fondazione Cenci Bolognetti, Department of Biology and Biotechnologies "Charles Darwin", Sapienza University of Rome, Rome, Italy.

~ Current address: Department of Stem Cell Biology, Murdoch Children's Research Institute, The Royal Children's Hospital, Parkville, Melbourne, Victoria, Australia.

[#] Current address: Department of Molecular Medicine, Sapienza University of Rome, Rome, Italy.

* corresponding author: alessandro.rosa@uniroma1.it

	Soma		Neurite	
	FUS^{WT}	FUS^{P525L}	FUS^{WT}	FUS^{P525L}
		UP		UP
CALM3	14282,78	16120,38	5316,86	7729,44
MAP1B	13029,50	15843,10	3829,03	5467,39
AP2B1	6220,62	7701,00	3782,13	4594,11
SYT1	2375,27	3226,03	415,82	1618,64
ACLY	3444,22	4448,06	1625,59	2696,47
APBA1	653,93	1093,99	129,02	381,57
PTEN	1532,79	2004,98	1066,19	1333,11
HuD	976,85	3969,72	224,61	908,96

	Soma		Neurite	
	$FMRP^{WT}$	$FMRP^{KO}$	$FMRP^{WT}$	$FMRP^{KO}$
		UP		UP
PPP3CA	1360,44	1498,87	646,10	825,37
PTEN	1574,18	1734,61	842,01	1152,77
PPP2R1A	3261,28	3995,29	2278,10	2757,04
MAP1B	6436,31	15143,71	3527,93	5118,23
AP2B1	193,53	738,39	2579,71	3799,79

		DOWN		DOWN
NCKAP1	5529,04	4341,67	3702,24	3341,71
PTK2	1290,02	840,65	748,06	589,16
ULK1	1216,13	941,37	734,47	501,21

	Soma		Neurite	
	FUS^{WT}	FUS^{WT} + SYN::HuD	FUS^{WT}	FUS^{WT} + SYN::HuD
		UP		UP
SYT1	2227,48	2483,59	459,33	1412,84
ULK1	898,19	1117,81	543,16	725,74
AGAP1	1402,68	1882,37	428,37	1071,73
CTBP1	1333,29	1672,00	1174,59	1487,78
DAB2IP	446,88	629,13	369,59	520,22
HUWE1	1596,93	2306,64	1664,14	1919,38
PLEC	165,66	285,29	165,25	239,09

		UP		DOWN
MYH10	2318,46	2783,83	6224,58	2738,76
PACS1	1830,39	2411,59	2837,37	2350,10

		DOWN		UP
AP2B1	5833,59	5323,63	4177,88	5072,81
CALM3	13394,13	10827,61	5873,19	9507,27
STXBP5	797,74	372,52	165,57	262,89

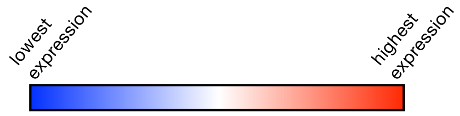

Supplementary Figure S1. Analysis of genes whose expression is altered in both soma and neurite compartments

The tables show the average counts by Nanostring (from three independent experiments) of the transcripts that change in abundance in both soma and neurite compartments in FUS^{P525L} (top left), $FMRP^{KO}$ (bottom left) or FUS^{WT} SYN::HuD (right) MNs compared with their respective controls. The heatmap is relative to each gene in the four samples: red, highest expression; blue, lowest expression. On the top of each table, UP indicates increased abundance, and DOWN decreased abundance, in the mutant or HuD overexpressing condition.

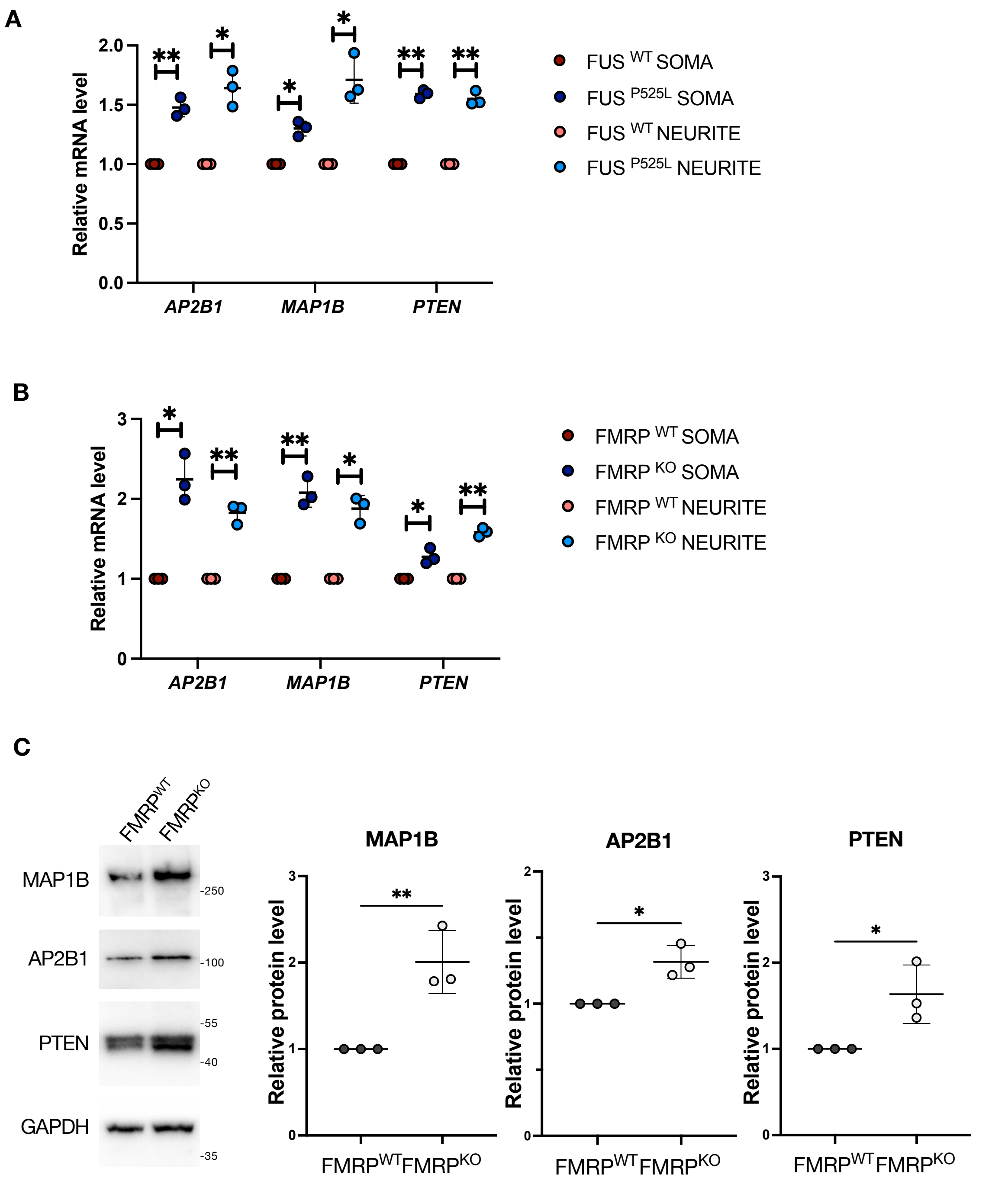

Supplementary Figure S2. Analysis of *AP2B1*, *PTEN* and *MAP1B* mRNA and protein levels

(A-B) Analysis of the mRNA levels of the indicated genes by real-time qRT-PCR in iPSC-derived spinal MNs. The graphs show the average from three independent differentiation experiments, error bars indicate the standard deviation (Student's t-test; unpaired; two tails; *p < 0.05; **p < 0.01). **(C)** Western blot analysis of the indicated genes protein levels in $FMRP^{WT}$ and $FMRP^{KO}$ iPSC-derived

spinal MNs. The molecular weight (kDa) is indicated on the right. The graphs show the average from three independent differentiation experiments, error bars indicate the standard deviation (Student's t-test; unpaired; two tails; *$p < 0.05$; **$p < 0.01$). GAPDH signal was used for normalization. Protein levels are relative to the FMRPWT sample for each experiment.

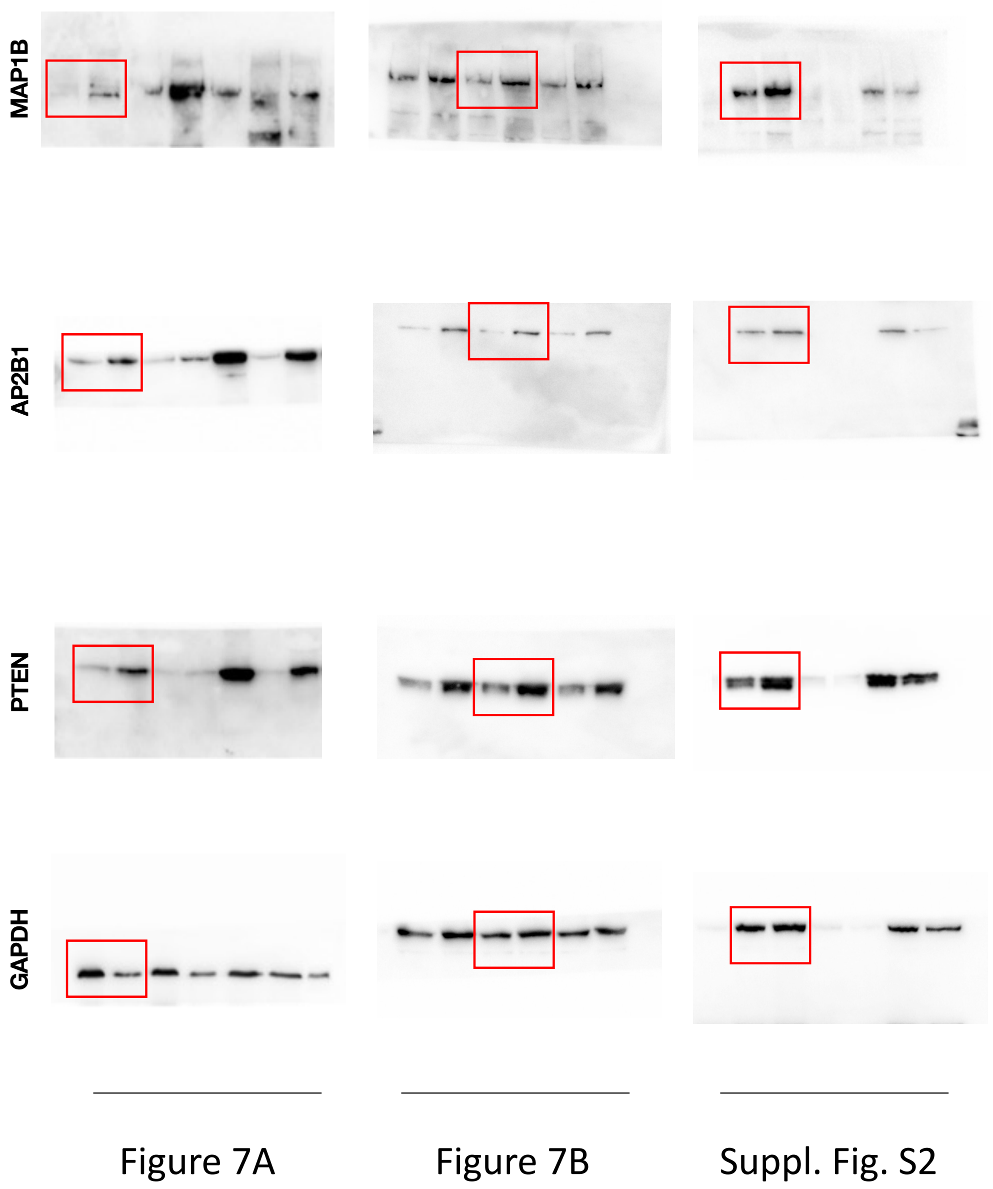

Supplementary Figure S3. Uncropped western blot images

Uncropped western blot images relative to the experiments showed in Figure 7 and Supplementary Figure S2, indicated with a red box.

Supplementary Table S1. Intersection between FUS and FMRP CLIP targets

Supplementary Table S2. GO term enrichment analysis of common FUS and FMRP targets

Supplementary Table S3. List of genes analyzed by digital color-coded molecular barcoding and other common targets

Supplementary Table S4. nCounter custom code set used in this study

Supplementary Table S5. GO term enrichment analysis of differentially expressed genes

Supplementary Table S6. Sequences of the PCR primers
