# Supplementary figures and images for: Digital color-coded molecular barcoding reveals dysregulation of common FUS and FMRP targets in soma and neurites of ALS mutant motoneurons
Source: Cell Death Discov. 2023 Jan 26;9:33. doi: 10.1038/s41420-023-01340-1 (PMC9879958; doi:10.1038/s41420-023-01340-1)

## Full length uncropped original western blots

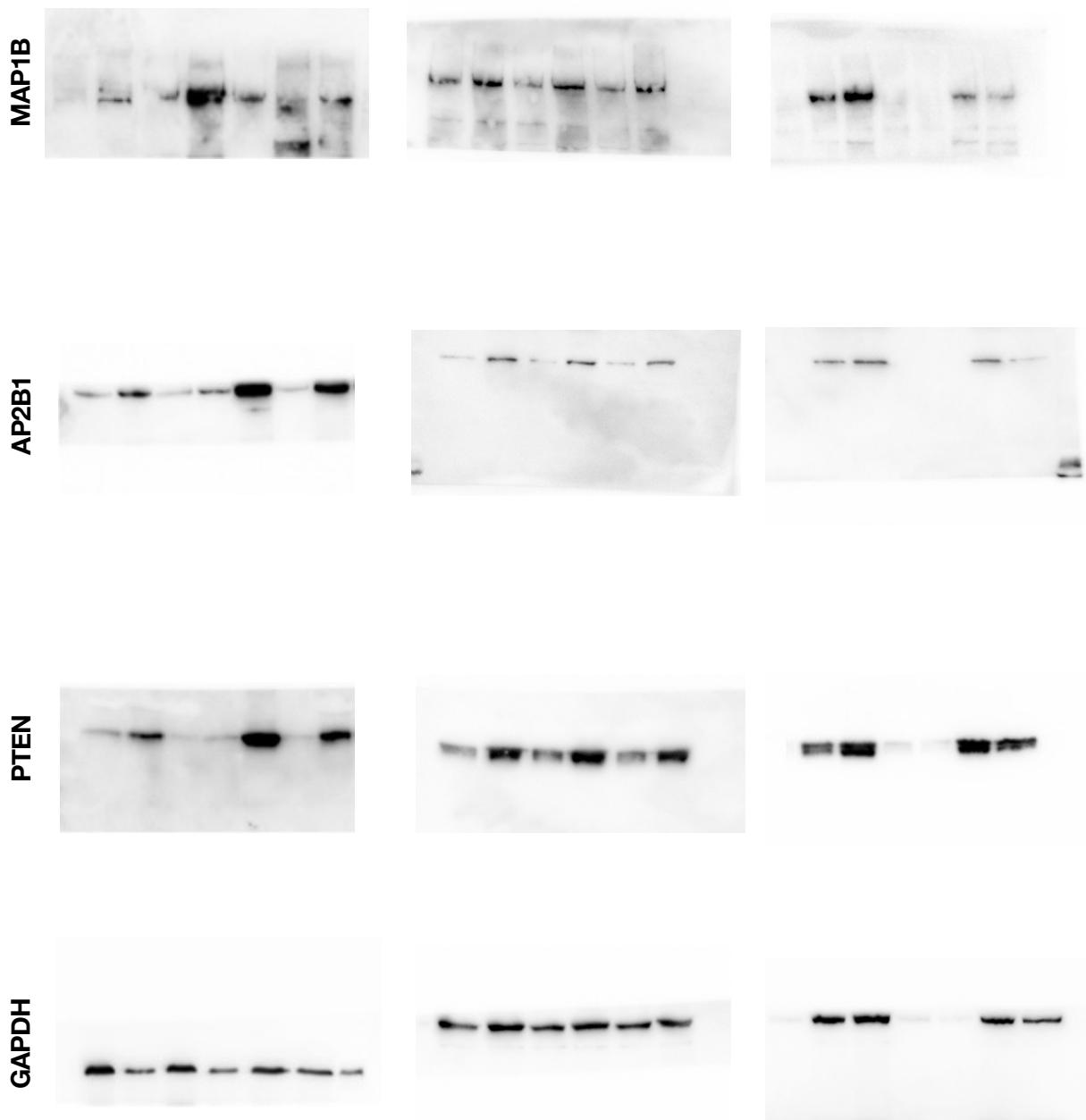

Figure 7A

Figure 7B

Suppl. Fig. S2

Supplement: Supplementary file 2 — Full lenght uncropped western blots [file 41420_2023_1340_MOESM2_ESM.pdf]
